# De-Ubiquitinating Enzymes USP21 Regulate MAPK1 Expression by Binding to Transcription Factor GATA3 to Regulate Tumor Growth and Cell Stemness of Gastric Cancer

**DOI:** 10.3389/fcell.2021.641981

**Published:** 2021-03-15

**Authors:** Qingqu Guo, Dike Shi, Lele Lin, Hongbo Li, Yunhai Wei, Baozhong Li, Dan Wu

**Affiliations:** ^1^Department of Gastrointestinal Surgery, The Second Affiliated Hospital, College of Medicine, Zhejiang University, Hangzhou, China; ^2^Department of Gastrointestinal Surgery, Huzhou Central Hospital, Huzhou, China; ^3^Department of Surgery, Anyang Tumor Hospital, Anyang, China

**Keywords:** gastric cancer, USP21, GATA3, MAPK1, de-ubiquitination, stemness

## Abstract

USP21 is a kind of deubiquitinating enzymes involved in the malignant progression of various cancers, while its role in gastric cancer (GC) and the specific molecular mechanism are still unclear. This study probed into the function of USP21 *in vitro* and *in vivo*, and discussed the regulatory mechanism of USP21 in GC *in vitro*. We reported that USP21 promoted GC cell proliferation, migration, invasion, and stemness *in vitro*, and regulated GC tumor growth and cell stemness in mice *in vivo*. USP21 stabilized the expression of GATA3 by binding to GATA3. Besides, GATA3 also regulated the expression of MAPK1 at the transcriptional level. A series of *in vitro* experiments testified that USP21 regulated the expression of MAPK1 by binding to transcription factor GATA3, thereby regulating the tumor growth and cell stemness of GC. Overall, this study identified a new USP21/GATA3/MAPK1 axis, which plays a pivotal role in promoting the malignant progression of GC and might provide a potential target for treatment.

## Introduction

Gastric cancer (GC) as one of the most common malignancies in the digestive system remains one of the top 10 cancer-related deaths in many Asian countries (Wu and Lin, 2020). Due to a lack of clinical symptoms in early GC, most patients are diagnosed at later stages with poor prognosis and low survival rate, and for patients with advanced GC, the average overall survival (OS) is even less than 12 months (Figueiredo et al., 2017; Padmanabhan et al., 2017; Zhang and Zhang, 2017). Surgical resection is currently considered to be the unique radical cure (Aurello et al., 2020), whereas it usually brings huge metabolic pressure to patients (Wu and Lin, 2020). As reported, molecular characterization of GC may bring us more effective therapeutic strategies, such as individualized therapies and novel clinical trial designs, which will eventually advance the medical management of the disease (Serra et al., 2019). However, the specific molecular mechanism underlying GC development described so far is not quite sufficient. Hence, an intensive study of the molecular mechanism of GC may be beneficial for further improvement of patient’s treatment and prognosis.

De-ubiquitinating enzymes (DUBs) can remove ubiquitin molecules from protein substrates and maintain their stability (Liu et al., 2016). The human genome encodes about 90 DUBs, of which 79 DUBs may possess catalytic activity (Urbe et al., 2012). DUBs can be classified into five families: ubiquitin C-terminal hydrolase (UCH), ubiquitin-specific protease (USP), ovarian tumor, Josephins (Machado–Joseph disease), and JAB1/MPN/Mov34 metalloenzymes (JAMM/MPN+). Membranes of the first four families are cysteine proteases, while the JAMM family is the family of zinc metalloproteases (Urbe et al., 2012). USP21 as an effective DUB can catalyze isopeptide bond hydrolysis between ubiquitin and histone H2A (Okuda et al., 2013). In tumor research, USP21 is noted to participate in the malignant processes of a variety of cancers. In non-small cell lung cancer, USP21 promotes tumor cell proliferation, migration, and invasion through the YY1/SNHG16 axis (Xu et al., 2020). In bladder cancer, USP21 is highly expressed and patients with high expression levels have poor survival, and USP21 can accelerate the proliferation and metastasis of bladder cancer cells via inhibiting EZH2 ubiquitination (Chen et al., 2017). While in hepatocellular carcinoma, USP21 binds to MEK2 and regulates the polyubiquitination at Lys48, thereby stabilizing MEK2 and up-regulating ERK1/2 to support sustained proliferation and oncogenic signals (Li et al., 2018). In basal-like breast cancer, USP21 can also regulate the cell cycle and paclitaxel sensitivity of cancer cells by deubiquitinating the transcription factor FOXM1 (Arceci et al., 2019). Besides, USP21 as a DUB plays an important role in regulating cell stemness. For instance, USP21 stimulates the stemness of pancreatic cancer cells by activating the Wnt pathway (Hou et al., 2019). USP21 interacts with Nanog protein in embryonic stem cells *in vivo* and *in vitro* to deubiquitylate the K48-type linkage of the ubiquitin chain of Nanog, thereby stabilizing Nanog and in turn maintaining the stemness of embryonic stem cells in mice (Liu et al., 2016). However, the molecular mechanism of USP21 in regulating GC cell stemness remains an open issue.

This study identified the expression pattern of USP21 in GC and clarified its role in regulating cell proliferation, metastasis, and cell stemness, along with possible molecular mechanisms further discussed. This research provides a previously unrecognized mechanism for the progression of GC and offers novel potential clues for GC therapy.

## Materials and Methods

### Bioinformatics Analysis

USP21 expression in The Cancer Genome Atlas (TCGA) –STAD database was searched through Gene Expression Profiling Interactive Analysis (GEPIA) database^1^. Downstream regulatory factors of GATA3 were predicted through the hTFtarget database^2^. The R package “clusterprofiler” was used to analyze the significantly activated Kyoto Encyclopedia of Genes and Genomes (KEGG) pathways of the potential downstream target genes of GATA3. Gene functional association analysis was performed on the STRING database^3^, and a protein-protein interaction (PPI) network was established and further visualized using the Cytoscape v3.7.1. Core value of each gene in the network was counted. The possible binding sites between MAPK1 promoter region and GATA3 were predicted through the JASPAR database^4^.

### Clinical Samples

GC tissue samples of 22 patients who underwent surgical resection from May to August in 2020 in the Second Affiliated Hospital of Zhejiang University School of Medicine were collected, and the matched adjacent non-tumor tissue was obtained from the part farthest (>5 cm) from the tumor in each excised specimen. All samples were immediately frozen in liquid nitrogen after excision and stored at -80°C. The included samples of patients were all diagnosed with clear pathology of GC. The patients did not receive preoperative radiotherapy or chemotherapy. All the patients were informed with the sample collection information, and signed informed consent. The project was approved by the Research Ethics Committee of the Second Affiliated Hospital of Zhejiang University School of Medicine.

### Cell Culture

Human normal gastric mucosal epithelial cell line GES-1 (BNCC353464) and GC cell lines AGS (BNCC309318), MKN-28 (BNCC338339), MKN-45 (BNCC337682), and MGC-803 (BNCC100665) were accessed from BeNa Culture Collection (BNCC, Beijing, China). The cells were cultured in Dulbecco’s modified Eagle’s medium (DMEM) (Corning) supplemented with 10% fetal bovine serum (FBS) (Gibco) at 37°C with 5% CO_2_.

### Plasmid Construction and Transfection

The USP21 or GATA3 coding sequence was cloned into the pENTER plasmid (ViGene Biosciences Inc., Rockville, MD, United States) to overexpress USP21 or GATA3. The Flag or Myc labeled-empty plasmid vector (pENTER) was purchased from Vigene. The small interfering RNA (siRNA) targeting USP21, GATA3, or MAPK1 was designed and synthesized by RiboBio (Guangzhou, China). All transfections were performed using Lipofectamine 2000 (Invitrogen, Carlsbad, CA, United States). For *in vivo* experiment, cells were seeded into a 6-well plate and cultured overnight, and an appropriate lentivirus-packing vector overexpressing USP21 synthesized by GenePharma (Shanghai, China) was added to the cells for infection.

### Real-Time Quantitative Polymerase Chain Reaction (qRT-PCR)

Total RNA was isolated from tissue and cell lines using RNeasy Mini Kit (QIAGEN), 1 μg of which was reverse-transcribed into complementary DNA (cDNA) using RevertAid First Strand cDNA Synthesis Kit (Thermo Scientific, United States). qRT-PCR was performed on the StepOne real-time PCR system (Thermo Fisher Scientific) using SYBR Green PCR kit (Takara Bio, Otsu, Japan). The 2^–ΔΔ*Ct*^ method was employed to calculate the relative gene expression normalized by GAPDH. The primer sequences are detailed in Table 1.

**TABLE 1:** Primer sequences in qRT-PCR.

**Gene**	**Forward (5′-3′)**	**Reverse (5′-3′)**
USP21	CAGGTCTGCCTGATGAACGG	GCTAAGTTGGTCCGAGATGGG
GATA3	GCCCCTCATTAAGCCCAAG	TTGTGGTGGTCTGACAGTTCG
MAPK1	TACACCAACCTCTCGTACATCG	TACACCAACCTCTCGTACATCG
CD44	CTGCCGCTTTGCAGGTGTA	CATTGTGGGCAAGGTGCTATT
CD133	AGTCGGAAACTGGCAGATAGC	GGTAGTGTTGTACTGGGCCAAT
GAPDH	CCCATCACCATCTTCCAGGAG	CTTCTCCATGGTGGTGAAGACG

### Western Blotting

Total proteins were extracted from GC cells using enhanced radioimmunoprecipitation assay (RIPA) lysis buffer (Thermo Scientific, Waltham, MA, United States), and the obtained proteins were quantified using bicinchoninic acid (BCA) protein assay kit (Beyotime, Shanghai, China). The proteins were separated by polyacrylamide gel electrophoresis (PAGE) and transferred onto a polyvinylidene fluoride (PVDF) membrane (Millipore, Bedford, MA, United States). Then, the membrane was blocked with 5% skimmed milk powder prepared with Tris-buffered saline +Tween-20 (TBST) for 2 h. The membrane and the following primary antibodies, including rabbit anti-USP21, anti-GATA3, anti-MAPK1, anti-Flag, anti-Myc, anti-E-cadherin, anti-α-catenin, anti-fibronectin, anti-GAPDH, were incubated at 4°C overnight. After the membrane was washed, it was then incubated with secondary antibody goat anti-rabbit IgG H&L for 1 h at room temperature. Protein detection was carried out by the enhanced chemiluminescence (ECL) detection system (Amersham, RPN2132). ChemiDoc imaging system (BioRad) was run to obtain western blotting images. All antibodies used here were ordered from Abcam (Cambridge, United Kingdom).

### MTT Assay

MTT assay was used to assess cell viability. The transfected cells (2 × 10^3^ cells/well) were seeded into a 96-well plate. After cultured at 37°C for 24, 48, 72, or 96 h, they were treated with 0.5 mg/mL MTT solution (Sigma-Aldrich, Shanghai, China) for 2 h. The absorbance at 490 nm was measured with a microplate reader (Bio-Rad, Hercules, CA, United States) to evaluate the number of viable cells.

### Colony Formation Assay

After 48 h of transfection, the cells were collected and counted. 2 × 10^3^ cells were placed in a 6-well plate and incubated in complete medium for 14 days until clear colonies were formed. The colonies were fixed with 4% paraformaldehyde and stained with 0.5% crystal violet. Each well was washed with sterile water to remove residual crystal violet and the colonies were counted under a microscope.

### Transwell Migration and Invasion Assays

A total of 5 × 10^4^ cells prepared in a serum-free medium were seeded into the top chamber of the Transwell chamber (uncoated with Matrigel) (Corning Life Sciences, Corning, NY, United States) to assess cell migration. The invasion assay was performed using the Transwell chamber coated with Matrigel (BD, Franklin Lakes, United States). Complete medium was placed in the bottom chamber in both assays. After incubated at 37°C with 5% CO_2_ for 24 h, the cells on the upper surface of the filter were wiped off with a cotton swab. The cells in the bottom chamber were fixed in 4% paraformaldehyde, stained with 0.1% crystal violet, and counted in three random fields under a microscope (Zeiss, Germany).

### Sphere Formation Detection

Cells (1 × 10^3^) were seeded into Ultra Low Attachment 6-well plates (Corning Incorporated Life Sciences, Acton, MA, United States) and cultured in DMEM supplemented with B27, N2, 10 ng/mL epidermal growth factor, and 10 ng/mL basic fibroblast growth factor (Millipore). After 5 days of incubation, the spheres formed were counted with a stereomicroscope (Olympus, Tokyo, Japan). The sphere-forming capability of cells was evaluated by selecting three random fields under the microscope to count the number of spheres (>50 μm). To determine tumor cell-related sphere formation in nude mice, the tumor tissue was cut into pieces and digested with trypsin. Next, the digested cells were dispersed and filtered with a filter and were then washed with phosphate-buffered saline (PBS). Afterward, mice tumor cell separation solution (120909, Tiandz, Beijing) was used to separate tumor cells following the instructions.

### Co-immunoprecipitation (Co-IP)

RIPA buffer (comprised of 0.5 mM EDTA; 10% glycerol; 20 mM Tris-Cl, pH 8.0; 100 mM NaCl; 1% protease inhibitor cocktail; 1 mm PMSF; 0.5% NP-40) was used to lyse cells, and the cell lysate was used for Co-IP assay. In short, the cell lysate was immunoprecipitated with anti-USP21 or anti-GATA3 antibodies at 4°C for 3 h and then incubated with A/G protein (Santa Cruz Inc.) overnight at 4°C. The protein A/G-Sepharose complex was washed three times with hydroxyethyl piperazine ethanesulfonic acid (HEPES) buffer. Finally, western blotting was performed for identification of the proteins.

### Chromatin Immunoprecipitation (ChIP) and PCR Assay

ChIP analysis was carried out using the EZ ChiP kit (Merck Millipore, Bedford, MA, United States) per the manufacturer’s protocol to determine the interaction between GATA3 and the MAPK1 promoter region. The cells were cross-linked with 1% formaldehyde at 37°C for 10 min, and the cross-linking was quenched with glycine at room temperature. Then, the cells were collected and sonicated to cut the DNA into fragments of 200 bp to 1 kb, and the cell debris was centrifuged at 4°C. The sample was then incubated with target antibodies overnight at 4°C to obtain co-immunoprecipitate. Following washing and de-crosslinking, RT-PCR was used to determine the enrichment of GATA3 on the promoter region of MAPK1.

### Dual-Luciferase Reporter Gene Assay

The MAPK1 promoter and GATA3 3′-untranslated region (UTR) were cloned into the pmirGlo reporter gene vector (Promega, Madison, WI, United States) and were validated through DNA sequencing. A KOD-Plus Mutagenesis Kit (Toyobo Biochemicals, Osaka, Japan) was used to mutate the presumed binding sites according to the protocols. Lipofectamine 2000 (Invitrogen) was employed for cell transfection in 6-well plates, and 48h later, a dual-luciferase reporter assay kit (Promega) was applied to detect luciferase activity following the manufacturer’s instructions.

### Xenograft Tumor *in vivo*

Twelve male BALB/c nude mice (4 weeks old) were randomly divided into two groups (6 mice/group). The AGS cells transfected with the oe-USP21 lentivirus-packaging vector were washed with PBS and injected subcutaneously into the armpit area of either side of the nude mice. Tumor volume was calculated every 2 days through the formula V = 0.5 × ab^2^(a: tumor length, b: tumor width). The BALB/c nude mice were obtained from the Institute of Laboratory Animals Science, CAMS & PUMC (Beijing, China), and all experiments involving mice were approved by the Institute of Biophysics, Chinese Academy of Sciences. The grading classification of mice tumors referred to the standard adopted by Hou et al. (2019).

### Hematoxylin-Eosin (H&E) Staining and Immunohistochemistry (IHC)

The tumor tissue from mice was fixed in formalin, embedded in paraffin, and cut into 6 μm sections for H&E staining and IHC. The tissue sections were rehydrated and taken for antigen retrieval. Then, the tissue sections were incubated with Ki67 antibody (Abcam, Cambridge, United Kingdom) at 4°C for 12 h. After washed several times, the sections were incubated with goat anti-rabbit IgG H&L (Abcam, Cambridge, United Kingdom) at room temperature for 1 h, and then exposed to 3,3’-diaminobenzidine (DAB) solution (Sigma, St. Louis, MO, United States) for observation.

### Statistical Analysis

Statistical analysis was conducted using SPSS 22.0 (IBM Corp. Armonk, NY, United States) and GraphPad Prism 6.0 Software (GraphPad Inc., San Diego, CA, United States). All measurement data were expressed as mean ± SD. *T*-test was used for comparison between two groups, while the analysis of variance (ANOVA) was used for pairwise comparison among multiple groups. *P* < 0.05 indicated that the difference was statistically significant.

## Results

### USP21 Is Highly Expressed in GC Tissue and Cells

**FIGURE 1:**
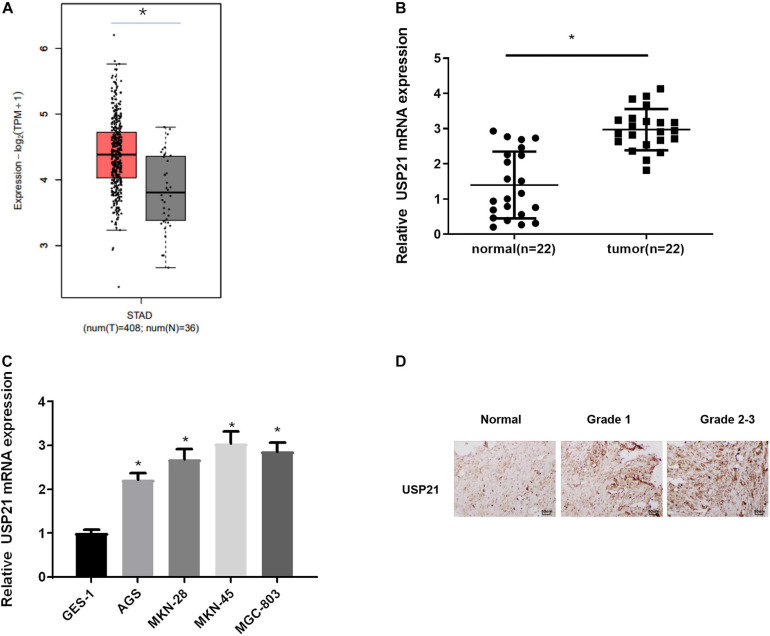
USP21 is highly expressed in GC tissue and cells. **(A)** The gene expression of USP21 in TCGA-STAD dataset (X-axis: sample type; Y-axis: relative expression value; The red box: tumor sample; The gray box: normal sample); **(B)** USP21 mRNA expression in clinical GC tissue and adjacent normal tissue (*n* = 22); **(C)** USP21 mRNA expression in human normal gastric epithelial cell line GES-1 and GC cell lines AGS, MKN-28, MKN-45, MGC-803; **(D)** IHC image shows the tissue location of USP21 in different tumor grades (400×); *p < 0.05.

To find out the role of DUBs in GC, the TCGA-STAD dataset was used to analyze the expression of USP21 in GC patients. It could be seen from Figure 1A that compared with normal tissue, USP21 was prominently highly expressed in GC tissue. qRT-PCR was performed to evaluate the mRNA expression of USP21 in clinical GC tissue and adjacent normal tissue from 22 GC patients. The results showed that the expression of USP21 in GC tissue was significantly higher than that in adjacent normal tissue (Figure 1B). Subsequently, the mRNA expression of USP21 determined in human normal gastric mucosal epithelial cell line and GC cell lines was presented in Figure 1C, showing that compared with normal gastric mucosal epithelial cell line GES-1, GC cell lines AGS, MKN-28, MKN-45, and MGC-803 had markedly increased USP21 mRNA. Figure 1D illustrated the results of IHC that tumor grading of GC was positively correlated with USP21 expression. Therefore, USP21 might play a regulatory role in the malignant progression of GC.

### USP21 Promotes the Proliferation, Migration, Invasion, and Stemness of GC Cells

**FIGURE 2:**
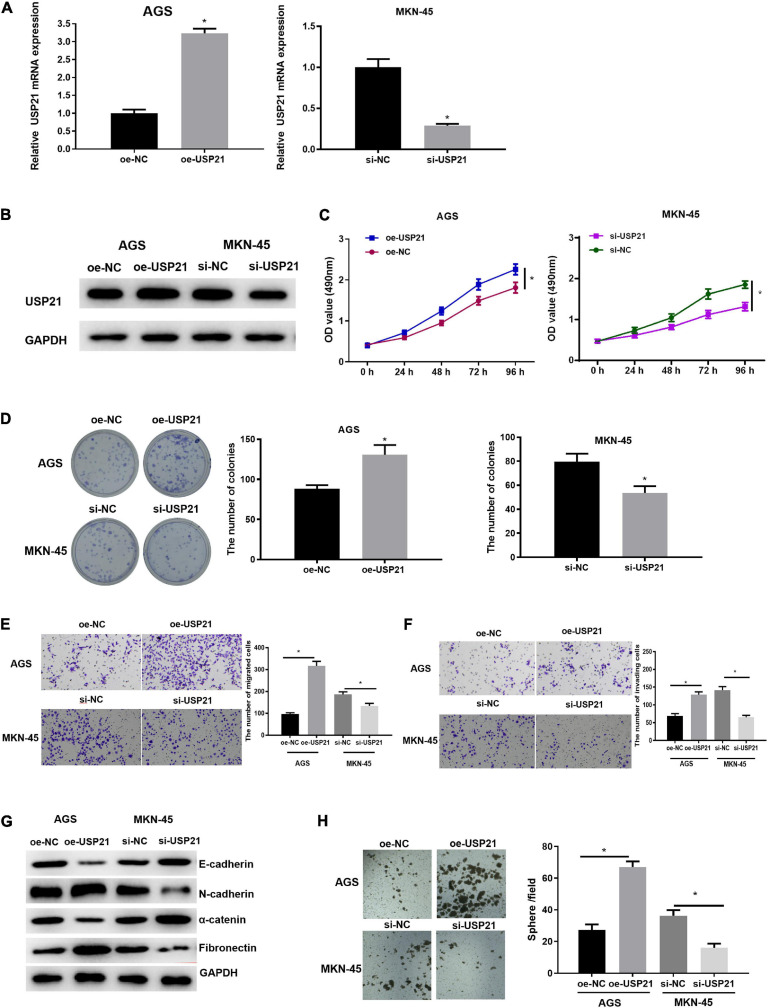
USP21 promotes the proliferation, migration, invasion, and stemness of GC cells. **(A)** The USP21 mRNA expression in each transfection group was detected by qRT-PCR; **(B)** The USP21 protein expression in each transfection group was measured by western blotting; **(C)** The effect of overexpression or inhibition of USP21 on the viability of GC cells was assessed via MTT assay; **(D)** The effect of overexpression or inhibition of USP21 on the proliferation of GC cells was measured through colony formation assay; **(E)** The effect of overexpression or inhibition of USP21 on the migration of GC cells was detected by Transwell assay (100×); **(F)** The effect of overexpression or inhibition of USP21 on the invasion of GC cells was measured via Transwell assay (100×); **(G)** The effect of overexpression or inhibition of USP21 on the expression of EMT marker proteins E-cadherin, N-cadherin, α-catenin, and fibronectin was evaluated by western blotting; **(H)** The number of spheres formed by cells treated with oe-USP21 or si-USP21; *p < 0.05.

Due to the abnormal expression of USP21 in GC, AGS and MKN-45 cell lines which, respectively, had relatively low and high USP21 expression in all tumor cells were selected to study the relationship between USP21 and GC progression. First, USP21-targeted plasmid (oe-USP21) was transfected into AGS cells for overexpression, while USP21 siRNA (si-USP21) was transfected into MKN-45 cells for gene silencing. Then, the mRNA and protein expression of USP21 were detected by qRT-PCR and western blotting, respectively. The results displayed that the expression of USP21 in AGS cells transfected with oe-USP21 was dramatically increased, while the expression of USP21 in MKN-45 cells transfected with si-USP21 was remarkably decreased (Figures 2A,B). To detect the effect of USP21 on cell proliferation, MTT and colony formation assays were performed. The results exhibited that overexpression of USP21 notably improved the viability and colony forming ability of AGS cells while inhibiting the expression of USP21 conspicuously reduced the viability and colony forming ability of MKN-45 cells (Figures 2C,D). Afterward, the role of USP21 in cell migration and invasion was evaluated through Transwell assay, and the data in Figures 2E,F showed that overexpression of USP21 accelerated the migration and invasion of AGS cells, whereas knockdown of USP21 pronouncedly reduced the migration and invasion of MKN-45 cells. Western blotting was conducted to test whether USP21 regulates epithelial-mesenchymal transition (EMT) by detecting the expression of several EMT marker proteins E-cadherin, N-cadherin, α-catenin, and fibronectin. It was observed that E-cadherin and α-catenin in the oe-USP21 group of AGS cells were significantly reduced, while N-cadherin and fibronectin were prominently increased. The opposite result appeared in the si-USP21 group of MKN-45 cells (Figure 2G). It is known that when grown in non-adherent serum-free medium, CSCs can complete self-renewal and then form spheres. To further determine the role of USP21 in the growth and maintenance of CSCs, the sphere-forming capability of AGS cells treated with oe-USP21 or of MKN-45 cells treated with si-USP21 was studied. As shown in Figure 2H, the sphere-forming capability of AGS cells treated with oe-USP21 was conspicuously elevated, while that of MKN-45 cells treated with si-USP21 was restrained. These results demonstrated that USP21 stimulated cell proliferation, migration, invasion, and stemness of GC cells.

### USP21 Can Interact With GATA3 *in vitro*

**FIGURE 3:**
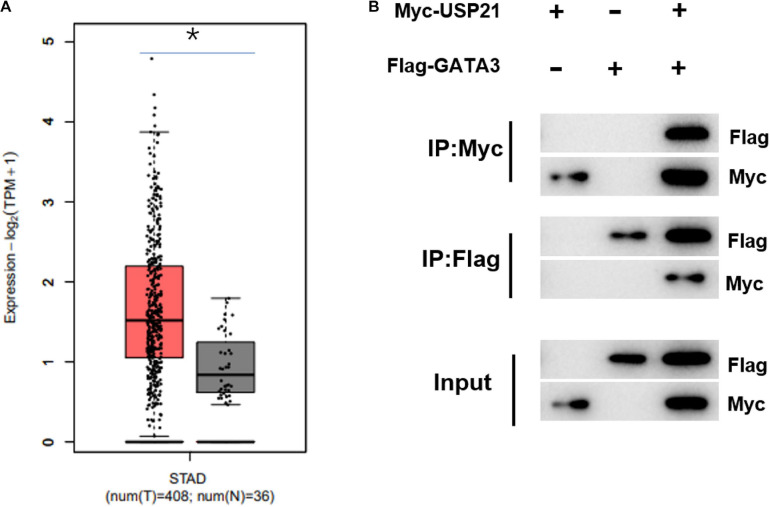
USP21 can interact with GATA3 *in vitro*. **(A)** The expression of GATA3 in TCGA-STAD dataset (X-axis: sample type; Y-axis: relative expression value; The red box: tumor sample; The gray box: normal sample); **(B)** Co-IP experiment verified the interaction between USP21 and GATA3; *p < 0.05.

Previous investigations testified that USP21 could participate in the progression of cancer. However, there have been few reports about whether the role of USP21 in GC correlates with GATA3. Here, it was found through gene expression data in TCGA-STAD that GATA3 was highly expressed in GC (Figure 3A). Immunoprecipitation was performed to study whether USP21 interacts with GATA3. Expression vectors containing Flag-tagged GATA3 (Flag-GATA3) and Myc-tagged USP21 (Myc-USP21) were co-transfected into HEK 293T cells. Myc-USP21 was immunoprecipitated with anti-Myc antibody and GATA3 was immunoprecipitated with anti-Flag antibody, and it was found that USP21 and Flag-GATA3 were co-immunoprecipitated (Figure 3B). These findings signified that USP21 and GATA3 could interact directly.

### MAPK1 Is the Transcriptional Target of GATA3

**FIGURE 4:**
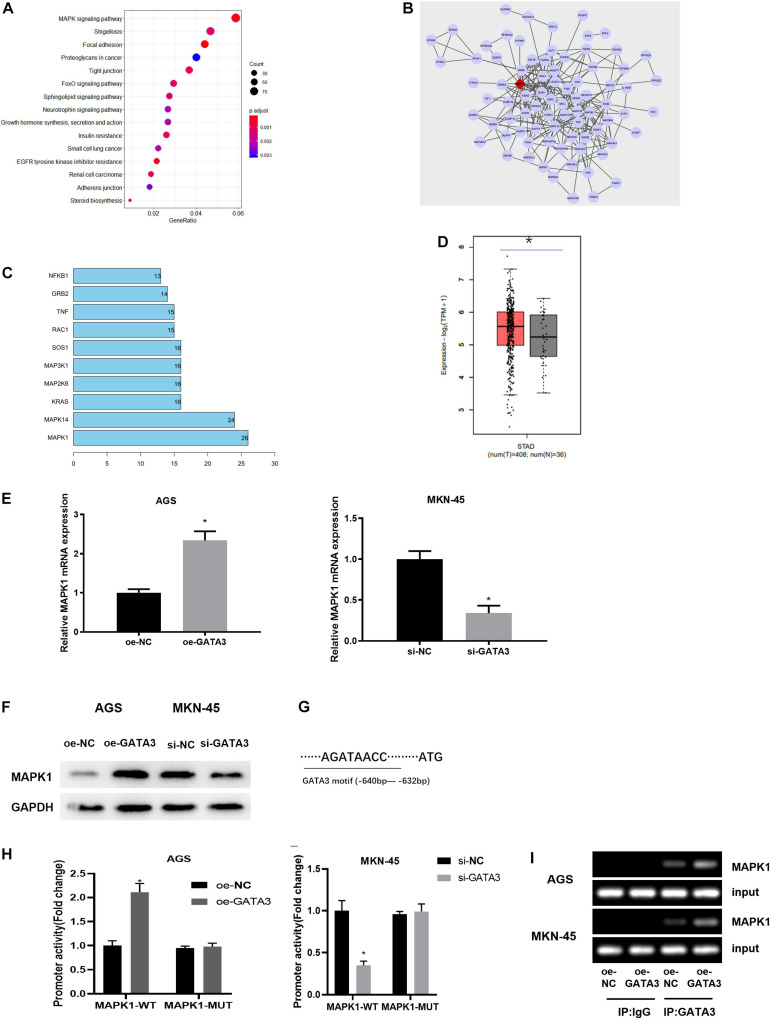
GATA3 regulates the expression of MAPK1. **(A)** Enrichment analysis of GATA3 potential downstream target genes (X-axis: GeneRatio; Y-axis: KEGG pathway; the right side: the color scale); **(B)** PPI analysis of potential target genes enriched in the MAPK signaling pathway (The line between genes in the figure indicates that there is an interaction between the two genes; The rhomboid gene is the target gene MAPK1); **(C)** Statistics of the core value of some genes in the PPI network; the number of interacted genes of a gene in the network is positively correlated with core value of the gene (X-axis: the core value; Y-axis: the gene name); **(D)** The relative expression of MAPK1 gene in TCGA-STAD dataset; **(E)** MAPK1 mRNA expression in AGS cells with overexpression of GATA3 or in MKN-45 cells with knockdown of GATA3; **(F)** MAPK1 protein expression in AGS cells with overexpression of GATA3 or in MKN-45 cells with knockdown of GATA3; **(G)** Binding site between GATA3 and MAPK1 promoter; **(H)** Dual-luciferase reporter gene assay was used to detect promoter activity; **(I)** ChIP was performed using the specific anti-GATA3 antibodies and IgG antibodies located on both sides of the MAPK1 promoter region, and the promoter region contains a putative GATA3 binding site; *p < 0.05.

To understand the downstream regulatory mechanism of GATA3, downstream regulatory genes of GATA3 were predicted on the hTFtarget database, and KEGG enrichment analysis was performed and revealed that the predicted target genes were mainly enriched in MAPK signaling pathway (Figure 4A). The genes enriched in the MAPK signaling pathway were further extracted and subjected to PPI analysis (Figure 4B). Meanwhile, core value of each gene in the PPI network was counted. It was found that the MAPK1 gene had the highest core value (Figure 4C), suggesting that MAPK1 may play the most important role. After further searching the expression of MAPK1 in TCGA-STAD dataset, MAPK1 was found to be highly expressed in GC tissue (Figure 4D). Taken together, it was speculated that MAPK1 might be a downstream target of GATA3.

To verify the above prediction, GATA3 overexpression plasmid (oe-GATA3) and GATA3 siRNA (si-GATA3) were constructed, and then they were, respectively, transfected into AGS cells and MKN-45 cells. qRT-PCR and western blotting manifested that overexpression of GATA3 dramatically increased the expression of MAPK1, while knockdown of GATA3 markedly reduced the expression of MAPK1 (Figures 4E,F). Given that GATA3 acted as a transcription factor and both GATA3 and MAPK1 were highly expressed in GC tissue, it was hypothesized that GATA3 might directly promote MAPK1 transcription. To validate the hypothesis, the first step was to produce a MAPK1 promoter containing a GATA3 binding site and establish a MAPK1 mutation using site-directed mutagenesis technology (Figure 4G). The results of dual-luciferase assay pointed out that elevation of GATA3 prominently increased the MAPK1 promoter activity in AGS cells, while silenced GATA3 reduced the promoter activity in MKN-45 cells, with no significant effect on the mutation of the MAPK1 promoter in both two groups (Figure 4H). ChIP assay was further conducted to determine the direct interaction between GATA3 and MAPK1 promoter. Figure 4I presented positive bands in the transfection group using GATA3 antibody. Therefore, it could be seen that the transcription factor GATA3 could interact with the MAPK1 promoter to stimulate the expression of MAPK1 in GC cells.

### USP21 Regulates the Expression of MAPK1 Through GATA3 and Affects Cell Proliferation, Migration, Invasion, and Stemness of GC

**FIGURE 5:**
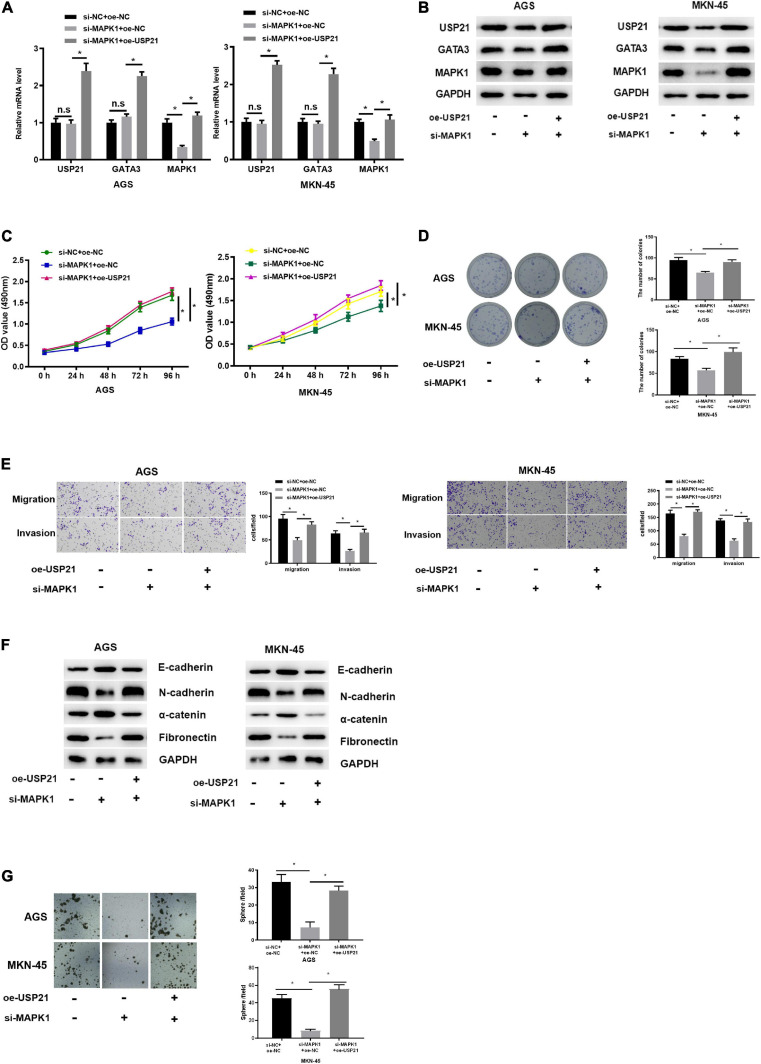
USP21 regulates the expression of MAPK1 through GATA3 and affects cell proliferation, migration, invasion, and stemness of GC. **(A)** qRT-PCR was used to detect the mRNA expression of USP21, GATA3, and MAPK1 in each group of cells; **(B)** Western blotting was performed to assess the protein expression of USP21, GATA3, and MAPK1 in each group of cells; **(C)** MTT assay was conducted to measure the viability of AGS cells and MKN-45 cells of each group; **(D)** Colony formation assay was carried on to determine the number of colonies of each group of AGS cells and MKN-45 cells; **(E)** Transwell assay was used to evaluate the migration and invasion of each group of AGS cells and MKN-45 cells; **(F)** Western blotting was conducted to evaluate the expression of EMT marker proteins E-cadherin, N-cadherin, α-catenin, and fibronectin in AGS cells and MKN-45 cells in each group; **(G)** The number of spheres formed by AGS cells and MKN-45 cells in different treatment groups; *p < 0.05.

Since USP21 stimulated cell proliferation, migration, invasion, and stemness of GC, and could stabilize the expression of GATA3 by deubiquitinating GATA3, while MAPK1 was the transcription target of GATA3, it was further verified whether USP21 could play its oncogenic effect in GC through the GATA3/MAPK1 axis. Three groups were set up for rescue experiments, namely: si-NC+ oe-NC, si-MAPK1+oe-NC, si-MAPK1+oe-USP21. The mRNA and protein expression of USP21, GATA3, and MAPK1 in each group of cells were analyzed by qRT-PCR and western blotting. The results demonstrated that the expression of MAPK1 was prominently decreased with knockdown of MAPK1, whereas the expression of USP21 and GATA3 had no obvious variation. While simultaneous knockdown of MAPK1 and overexpression of USP21 could conspicuously increase the expression of GATA3 and MAPK1 (Figures 5A,B), indicating that USP21 could regulate MAPK1 through GATA3. Cell proliferation in each group was measured and it was found that si-MAPK1 could restrain the proliferation of AGS cells and MKN-45 cells, while simultaneous knockdown of MAPK1 and overexpression of USP21 could reverse the inhibitory effect of si-MAPK1 on cell proliferation (Figures 5C,D). Transwell and western blotting were performed to evaluate EMT-related proteins E-cadherin, N-cadherin, α-catenin, and fibronectin in AGS cells and MKN-45 cells, thereby studying cell migration and invasion in each group. It could be seen from Figures 5E,F that after knocking down MAPK1, the migratory and invasive abilities of cells decreased pronouncedly, the E-cadherin and α-catenin in the cells increased significantly, and the N-cadherin and fibronectin decreased notably, while overexpression of USP21 partially inhibited these effects. Besides, it was found that si-MAPK1 conspicuously restrained the sphere-forming capability of AGS cells and MKN-45 cells, yet simultaneous transfection of si-MAPK1 and oe-USP21 dramatically improved the sphere-forming capability of cells (Figure 5G). These results indicated that USP21 regulated the expression of MAPK1 through GATA3 and then affected the proliferation, migration, invasion, and stemness of GC cells.

### USP21 Is Involved in Tumor Growth and Cell Stemness of GC *in vivo*

**FIGURE 6:**
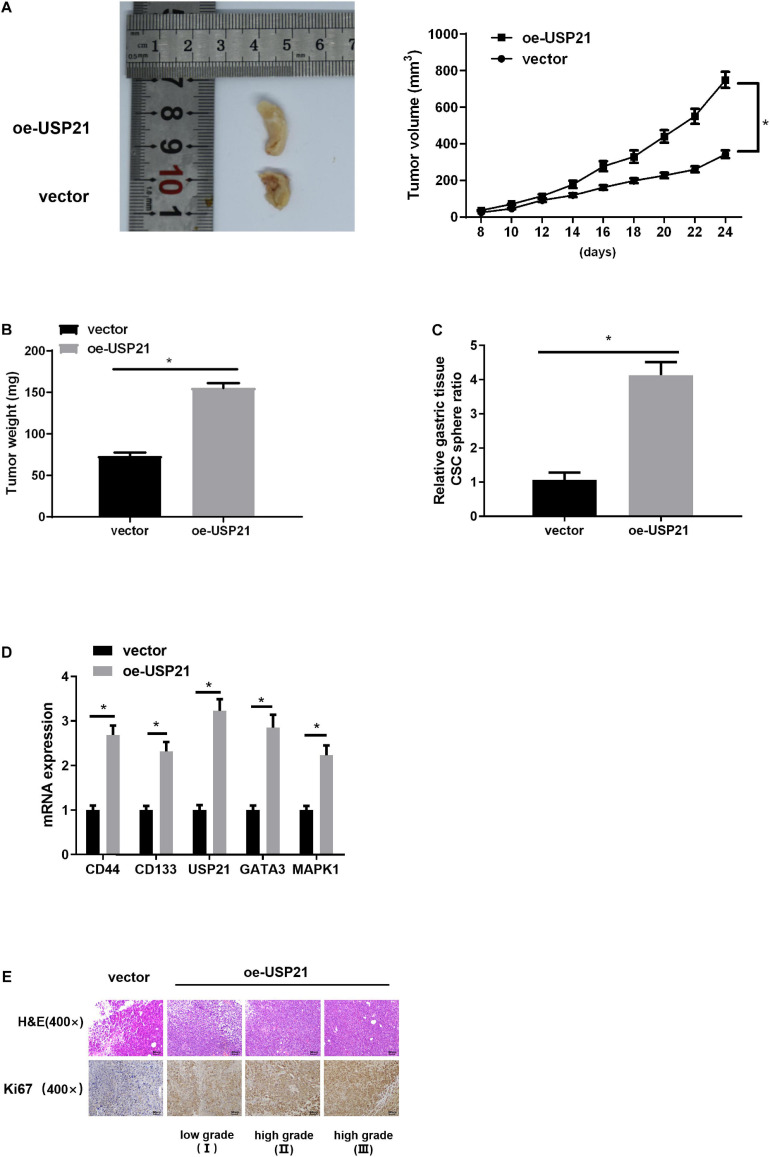
USP21 is involved in tumor growth and cell stemness of GC *in vivo*. **(A)** The effect of USP21 overexpression on tumor volume; **(B)** The effect of USP21 overexpression on tumor weight; **(C)** The effect of USP21 overexpression on cell sphere formation; **(D)** qRT-PCR was used to detect the influence of USP21 overexpression on the expression of CSC markers CD44, and CD133 as well as on the expression of USP21, GATA3, and MAPK1; **(E)** The expression of USP21 and Ki-67 in different grade of tumor tissue of mice in the control group and the oe-USP21 group; *p < 0.05.

Mouse xenograft models were established to further analyze the effect of USP21 on tumor growth *in vivo*. AGS cells overexpressing USP21 were subcutaneously injected into the collected BALB/c nude mice, and then the tumorigenic effect of USP21 *in vivo* was examined. Compared with the negative control group, the tumor weight and volume in the USP21 overexpression group were dramatically increased (Figures 6A,B). Besides, it was apparent in Figure 6C that overexpression of USP21 markedly increased the ratio of CSC spheres. The expression of CSC markers CD44 and CD133 in mice GC tissue was evaluated by qRT-PCR. The results disclosed that overexpression of USP21 remarkably elevated the expression levels of CD44 and CD133 in GC tissue (Figure 6D), indicating that overexpression of USP21 enhanced the stemness of GC cells. It was also found that in the tumor tissue of the oe-USP21 group, USP21, GATA3, and MAPK1 were all pronouncedly highly expressed. Evaluation of cell proliferation in nude mice by H&E staining and IHC revealed that Ki-67-positive cells in tumors of the oe-USP21 group were notably more than those in the control group, and the tumor grade was positively correlated with the number of Ki-67-positive cells as well as the content of USP21 (Figure 6E). Taken these findings together, it could be seen that USP21 regulated tumor growth and cell stemness of GC *in vivo*.

## Discussion

The ubiquitin system regulates a variety of biological processes including cancer progression by changing the ubiquitination state of protein substrates, which is achieved by ubiquitin ligase and DUBs affecting ubiquitin of the substrate to change its stability, activity, localization, and interaction (Gallo et al., 2017; Saldana et al., 2019). Studies found that several members of the DUB family are involved in GC carcinogenesis. For example, USP28 restrains the proliferation and invasion of GC cells (Zhao et al., 2018). USP42 is highly expressed in GC tissue and conspicuously associated with the tumor size, TNM staging, lymph node metastasis, and OS rate of GC patients, while inhibition of USP42 can induce G0/G1 block and suppress cell proliferation and invasion (Hou et al., 2016). USP3 facilitates cell migration and invasion by interacting with SUZ12 in GC and deubiquitinating (Wu et al., 2019). All these suggest that DUBs play an important role in the progression of GC.

In this study, USP21, another member of the USP subfamily of DUBs, was reported to be highly expressed in GC tissue and cells. The expression trend is consistent with that in breast cancer (Arceci et al., 2019) and colorectal cancer (Yun et al., 2020). Previous research found that the nuclear localization of USP21 in pancreas cancer is positively correlated with advanced tumor grades and expression levels, and overexpression of USP21 promotes cell stemness (Hou et al., 2019). Thus far, a study (Peng et al., 2016) proved that USP21 can accelerate cell growth, invasion, and stemness of renal cell carcinoma. In this study, it was also found that in GC tissue, the expression of USP21 was positively correlated with the grade of GC. Accordingly, USP21 might also have the function of regulating the stemness of GC cells. Thence, *in vitro* cell experiments were performed and it was found that overexpression of USP21 promoted cell proliferation, migration, invasion, EMT, and stemness, whereas inhibiting its expression posed opposite effects. Furthermore, it was also proved in nude mice that overexpression of USP21 stimulated the tumor growth and cell stemness of GC *in vivo*. Considering all of this evidence, it could be seen that USP21 acts as an oncogene in GC.

USP21 is proven to deubiquitinate TCF7 (Hou et al., 2019), YY1 (Xu et al., 2020), FOXM1 (Arceci et al., 2019), EZH2 (Chen et al., 2017), RIG-1 (Fan et al., 2014), Fra-1 (Yun et al., 2020), IL-8 (Peng et al., 2016), and other genes to stabilize their expression. This study testified that USP21 in GC cells could bind to GATA3 to regulate its expression. Then, the downstream regulatory factors of GATA3 were predicted, and MAPK1 was identified. GATA3 could interact with the MAPK1 promoter and mediate its expression, which was confirmed by ChIP and dual-luciferase reporter gene assays.

It is reported that regulation of MAPK pathway affects the stemness of cancer cells (Otte et al., 2019; da Cunha Jaeger et al., 2020; Luk et al., 2020). MAPK1, a key regulatory gene of the MAPK signaling pathway, was substantiated here whether USP21 regulates MAPK1 by combining with GATA3 and thus affects the cell stemness of GC. Functionally, it was disclosed that knocking down MAPK1 inhibited cell proliferation, migration, invasion, and stemness of GC as well as the expression of N-cadherin and fibronectin in the cells, while promoted the expression of E-cadherin and α-catenin. Nevertheless, overexpression of USP21 in cells with MAPK1 knockdown reversed this effect, indicating that the oncogenic effect of USP21 was achieved through the GATA3/MAPK1 axis.

This study clarified that USP21 as an oncogene facilitated cell proliferation, migration, invasion, and stemness of GC. What stood out in this study was that this oncogenic effect of USP21 was mediated through the GATA3/MAPK1 axis. In conclusion, this research established, for the first time, the role of the USP21/GATA3/MAPK1 axis in GC, providing a new mechanism for the pathological function of GC and having potential significance for the development of new targeted therapies.

## Data Availability Statement

All datasets generated for this study are included in the article/supplementary material, further inquiries can be directed to the corresponding author/s.

## Ethics Statement

All the patients were informed with the sample collection information and informed consent was obtained. The project was approved by the Research Ethics Committee of the Second Affiliated Hospital of Zhejiang University School of Medicine. All experiments involving mice were approved by the Institute of Biophysics, Chinese Academy of Sciences.

## Author Contributions

All authors contributed to the data analysis, drafting and revising the article, gave final approval of the version to be published, and agreed to be accountable for all aspects of the work.

## Conflict of Interest

The authors declare that the research was conducted in the absence of any commercial or financial relationships that could be construed as a potential conflict of interest.

## Footnotes

1 http://gepia2.cancer-pku.cn/#index

2 http://bioinfo.life.hust.edu.cn/hTFtarget#!/

3 https://string-db.org

4 http://jaspar.genereg.net
